# Vitamin D and the Thyroid: A Critical Review of the Current Evidence

**DOI:** 10.3390/ijms24043586

**Published:** 2023-02-10

**Authors:** Mirjana Babić Leko, Iva Jureško, Iva Rozić, Nikolina Pleić, Ivana Gunjača, Tatijana Zemunik

**Affiliations:** Department of Medical Biology, University of Split School of Medicine, 21 000 Split, Croatia

**Keywords:** vitamin D, thyroid, thyroid stimulating hormone (TSH), anti-thyroid antibodies, thyroid hormones, autoimmune thyroid diseases

## Abstract

Vitamin D is necessary for the normal functioning of many organs, including the thyroid gland. It is, therefore, not surprising that vitamin D deficiency is considered a risk factor for the development of many thyroid disorders, including autoimmune thyroid diseases and thyroid cancer. However, the interaction between vitamin D and thyroid function is still not fully understood. This review discusses studies involving human subjects that (1) compared vitamin D status (primarily determined by serum calcidiol (25-hydroxyvitamin D [25(OH)D]) levels) with thyroid function assessed by thyroid stimulating hormone (TSH), thyroid hormones, and anti-thyroid antibody levels; and (2) evaluated the effect of vitamin D supplementation on thyroid function. Due to the many inconsistencies in the results between the studies, it is still difficult to draw a definite conclusion on how vitamin D status affects thyroid function. Studies in healthy participants observed either a negative correlation or no association between TSH and 25(OH)D levels, while the results for thyroid hormones showed high variability. Many studies have observed a negative association between anti-thyroid antibodies and 25(OH)D levels, but equally many studies have failed to observe such an association. Regarding the studies that examined the effect of vitamin D supplementation on thyroid function, almost all observed a decrease in anti-thyroid antibody levels after vitamin D supplementation. Factors that could contribute to the high variability between the studies are the use of different assays for the measurement of serum 25(OH)D levels and the confounding effects of sex, age, body-mass index, dietary habits, smoking, and the time of year when the samples were collected. In conclusion, additional studies with larger numbers of participants are needed to fully understand the effect of vitamin D on thyroid function.

## 1. Introduction

This review aims to investigate the relationship between vitamin D and thyroid function. Since vitamin D has an important role in normal thyroid function, studies that are trying to understand the complex background of vitamin D and thyroid interaction are of utmost importance. In this review, we included studies involving human subjects that (1) compared vitamin D status with thyroid function, and (2) evaluated the effect of vitamin D supplementation on thyroid function. A literature search was performed by two independent researchers and completed on December 31, 2022. It was performed in Medline using the keywords “vitamin D”, “thyroid”, “25-hydroxyvitamin D”, and “25(OH)D” and was not limited by publication date.

## 2. Vitamin D

Different types of vitamin D are fat-soluble secosteroids (vitamin D1-D5). The most important types of vitamin D for humans are vitamin D3 (cholecalciferol) and vitamin D2 (ergocalciferol). Most vitamin D is synthesized in the skin after exposure to sunlight (vitamin D3), while only 5–10% is taken from food (vitamin D2 and D3) [1]. Exposure of skin to sunlight causes the transformation of 7-dehydrocholesterol into vitamin D3. Vitamin D3 is then transformed in the liver by the action of vitamin D 25-hydroxylase into 25-hydroxyvitamin D (25(OH)D, also known as calcidiol or calcifediol). The active form of vitamin D3, 1α,25-dihydroxyvitamin D (1,25(OH)2D, also known as 1,25-dihydroxycholecalciferol, 1α,25-dihydroxyvitamin D3 or calcitriol), is produced from the 25(OH)D in the kidneys by the action of the enzyme 1α-hydroxylase encoded by the *CYP27B1* gene [2]. Vitamin D status is mainly determined by measuring serum 25(OH)D.

The main role of calcitriol (the active form of vitamin D3) is the regulation of calcium and phosphate concentrations. Calcitriol increases intestinal and renal absorption of calcium and phosphate. It is also crucial for bone mineralization [3]. Additionally, calcitriol is involved in the regulation of cell growth, and immune and neuromuscular functions. Its anticancer and immunosuppressive effects have also been shown [4,5].

Calcitriol binds to the vitamin D receptor (VDR) which belongs to the nuclear receptor superfamily. After calcitriol binding, VDR dimerizes with the retinoid X receptor (RXR), translocates to the nucleus, and binds to vitamin D response elements within DNA [6]. VDR is involved in the regulation of the expression of more than 1000 genes [7,8] and is found in almost all tissues [9].

## 3. Thyroid Function

The thyroid gland synthesizes thyroid hormones that are crucial for the normal functioning of physiological systems. The hypothalamus-pituitary-thyroid (HPT) axis orchestrates thyroid hormone synthesis by feedback mechanisms. In other words, when the levels of thyroid hormones decrease, the hypothalamus synthesizes thyrotropin-releasing hormone (TRH). TRH stimulates the anterior pituitary, causing an increase in thyroid-stimulating hormone (TSH) secretion. Finally, TSH stimulates thyrocytes which increases the production of thyroid hormones [10]. The synthesis of thyroid hormones requires the active uptake of iodide through sodium/iodide symporter (NIS), production of thyroglobulin (Tg), and iodination of Tg by the enzyme thyroid peroxidase (TPO). When Tg is proteolyzed, thyroid hormones triiodothyronine (T3) and thyroxine (T4) are released. Although the thyroid releases more T4 than T3 (in a ratio of approximately 14:1) [11], the majority of T4 converts to T3 in the tissues [12]. This conversion is mediated by the enzymes type 1 and type 2 iodothyronine deiodinases (Dio1 and Dio2) [12]. When secreted in plasma, thyroid hormones are bound to plasma proteins; only 0.03% of thyroid hormones are in an unbound or free form (fT4 and fT3) that is biologically active [13].

## 4. Vitamin D in Thyroid Disorders

### 4.1. Vitamin D in Autoimmune Thyroid Diseases

Autoimmune thyroid diseases are characterized by an immune attack of the thyroid gland. These conditions are the most common autoimmune disorders in general, with a prevalence of approximately 5% [14]. Hashimoto’s thyroiditis, characterized by hypothyroidism, and Graves’ disease, characterized by hyperthyroidism, are the two main types of autoimmune thyroid diseases. Both conditions are T-cell-mediated autoimmune disorders characterized by thyroid lymphocytic infiltration [14].

Vitamin D supplementation has been shown to be beneficial in animal models of Graves’ disease [15] and thyroiditis [16]. To date, many human studies have also been conducted to evaluate the role of vitamin D in autoimmune thyroid diseases. Genetic studies have found that polymorphisms in *VDR* and other genes involved in vitamin D signaling are associated with an increased risk of autoimmune thyroid diseases [17,18,19]. A recent meta-analysis by Štefanić and Tokić, which included 25 studies (2695 cases with Hashimoto’s thyroiditis and 2263 controls), detected significantly decreased levels of 25(OH)D in patients with Hashimoto’s thyroiditis [20]. Additionally, a meta-analysis by Xu et al., which included 26 studies (1748 cases with Graves’ disease and 1848 controls), noted that patients with Graves’ disease were more likely to be vitamin D deficient [21]. However, a recent meta-analysis by Taheriniya et al., which included 42 studies analyzing patients with autoimmune thyroid diseases (1886 with autoimmune thyroid disease, 372 with hypothyroidism, 1375 with Hashimoto’s thyroiditis, and 604 with Graves’ disease), showed that vitamin D deficiency is associated with the development of autoimmune thyroid diseases, Hashimoto’s thyroiditis, and hypothyroidism. For Graves’ disease, however, association with vitamin D levels was shown only among older subjects [22].

In addition to its association with autoimmune thyroid diseases, vitamin D deficiency has also been observed in other autoimmune diseases such as multiple sclerosis, diabetes mellitus, systemic lupus erythematosus, and others [23,24]. Both VDR and 1α-hydroxylase are expressed in immune cells, T and B lymphocytes, dendritic cells, neutrophils, and monocytes [25,26]. Therefore, these cells can produce calcitriol, the active form of vitamin D3 [27]. Vitamin D can modulate the activity of various immune system cells and is involved in the regulation of the immune system. Vitamin D inhibits the production of proinflammatory cytokines such as IL-6, IL-8, IL-9, IL-12, IFN-γ, and TNF-α. It also enhances the production of anti-inflammatory cytokines such as IL-10, IL-5, and IL-4. The overall effect of vitamin D is considered to be anti-inflammatory [26]. Despite the numerous studies conducted to clarify the role of vitamin D in the development of autoimmune thyroid diseases, it is still unclear whether vitamin D deficiency is an important factor in the pathogenesis or the consequence of autoimmune thyroid diseases [28].

### 4.2. Vitamin D in Thyroid Cancer

The incidence of thyroid cancer is increasing. In 2017, 255,490 new cases of thyroid cancer were detected worldwide, while only 95,030 new cases were detected in 1990 [29]. In thyroid cancer, both follicular thyroid cells and neuroendocrine cells can be affected. Differentiated thyroid cancer (papillary thyroid cancer, Hurthle cell thyroid cancer, and follicular thyroid cancer), poorly differentiated thyroid cancer, and anaplastic (undifferentiated) thyroid cancer arise from thyroid follicular cells. Medullary thyroid cancer is caused by malignant changes in parafollicular neuroendocrine cells [30]. Differentiated thyroid carcinomas are the most common types of thyroid cancer with 85% of all cases having papillary thyroid cancer [29,30,31].

Both in vitro and in vivo studies have shown a beneficial effect of vitamin D in treating thyroid cancer. In vitro studies have shown that calcitriol and its analogue (MART-10) can inhibit the proliferation [32] and metastatic potential [33] of anaplastic thyroid carcinoma cells, respectively. In addition, the expression levels of VDR and other genes involved in vitamin D signaling are increased in malignant thyroid cells [34,35,36], suggesting a potential antitumor response of vitamin D in cancer [35]. In vivo studies have shown that treatment with calcitriol reduced tumor size in both mouse models of follicular thyroid cancer [37] and metastatic follicular thyroid cancer [38].

As for human studies, in a large randomized clinical trial involving 25,871 participants (including patients with lung, breast, prostate, and colorectal cancer), vitamin D3 supplementation was shown to reduce the risk of developing advanced cancer in individuals without a diagnosis at the beginning of the study [39]. Regarding thyroid cancer, a meta-analysis by Zhao et al. showed that vitamin D deficiency may be a risk factor for thyroid cancer [40]. Some studies, however, have found no association between vitamin D status and risk of developing thyroid cancer [41,42].

## 5. The Effect of Vitamin D on Secretion of TSH, Thyroid Hormones and Anti-Thyroid Antibodies

### 5.1. Evidence from Animal/Cell Models

There are several insufficiently understood mechanisms by which vitamin D might alter the levels of TSH and thyroid hormones (reviewed in [43]). Experimental studies have shown that vitamin D has a direct effect on Dio2, the enzyme necessary for the conversion of T4 into T3 in target tissues. Specifically, the administration of vitamin D3 in diabetic rats leads to an increase in Dio2 expression levels in the liver and brain and, consequently, an increase in fT3 levels and a decrease in fT4 levels [44]. However, the thyroid physiology in VDR knockout mice did not show significant changes, and the mice had only a moderate reduction in TSH levels [45]. In vitro studies have shown that calcitriol administration suppressed TSH-stimulated adenylyl cyclase activity [46] and iodide uptake [46,47], while a study in rat pituitary cells has shown that calcitriol administration increases TRH-induced TSH release [48]. These data indicate that vitamin D could have both central and peripheral effects on the release of TSH and thyroid hormones. However, further experimental studies are needed to clarify the underlying mechanisms.

### 5.2. Evidence from Human Studies

#### 5.2.1. Observational Studies

**Figure 1:**
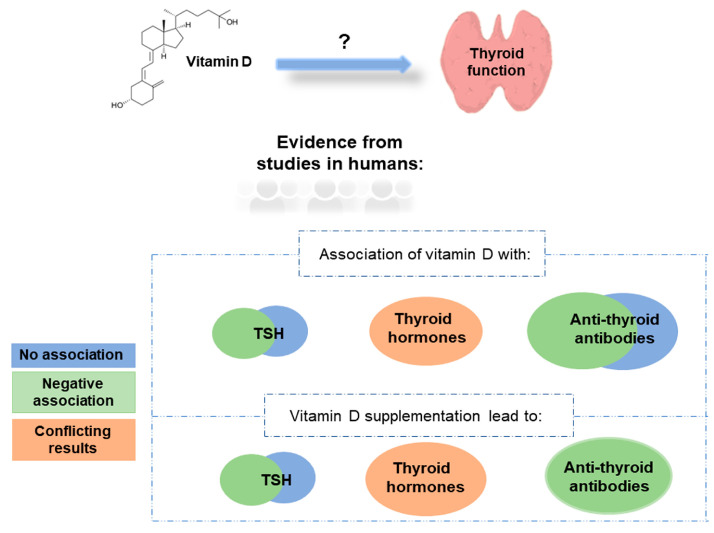
Association of vitamin D with thyroid function (evidence from studies in humans).

Many studies have linked 25(OH)D levels with the levels of TSH, thyroid hormones, and anti-thyroid antibodies (Table 1 and Figure 1). Studies in healthy participants mainly observed either a negative correlation [49,50] or no association [51,52] between TSH and 25(OH)D levels, and the same pattern was observed in studies involving patients with thyroid cancer (Table 1). Regarding thyroid hormones, conflicting results were observed in healthy participants with either positive [53], negative [54], or no association with 25(OH)D levels detected [55]. Studies involving patients with autoimmune thyroid diseases have also produced conflicting results. Two-thirds of the studies observed no association, while one-third of the studies detected a negative association between TSH and 25(OH)D levels (summarised in Table 1). On the other hand, most of the studies involving patients with autoimmune thyroid diseases did not observe an association between thyroid hormones and 25(OH)D levels. Regarding anti-thyroid antibodies, most of the studies observed a negative association between anti-thyroid antibody levels (anti-thyroid peroxidase antibody [TPOAb], anti-thyroglobulin antibody [TgAb], or TSH receptor antibody [TSHRAb]) and 25(OH)D levels, but many studies also failed to observe such an association (Table 1).

#### 5.2.2. Randomised Controlled Trials

Vitamin D deficiency is widespread, and it is estimated that approximately 40% of Europeans are vitamin D deficient with 13% being severely deficient [106]. Serum 25(OH)D levels below 50 nmol/L (or 20 ng/mL) and 30 nmol/L (or 12 ng/mL) are considered vitamin D deficiency and severe deficiency, respectively [107]. Therefore, many scientists and physicians recommend vitamin D supplementation. An international consensus on the optimal concentration for vitamin D supplementation has not yet been reached. In many countries, a daily vitamin D supplementation ranging from 400 to 2000 IU (10–50 μg) is recommended [107]. However, it is important to take into account that the prevalence of vitamin D deficiency varies in different ethnic groups. A study in Europeans showed that European Caucasians are less vitamin D deficient than non-white individuals [106]. Additionally, a study conducted in the USA showed that White individuals show lower rates of vitamin D deficiency than Black individuals [108]. Vitamin D deficiency is considered a risk factor for various diseases. It can lead to loss of bone density and increase the risk of fractures, osteoporosis, osteomalacia, and rickets in children [109]. In addition to autoimmune diseases, vitamin D deficiency has also been associated with cardiovascular diseases, neuropsychiatric disorders, and cancer [23,24]. Vitamin D deficiency may even contribute to the severity of COVID-19 [110].

Studies investigating the effect of vitamin D (cholecalciferol) supplementation on thyroid function were mostly conducted in patients with autoimmune thyroid diseases (Table 2). In almost all studies, vitamin D supplementation caused a significant reduction in anti-thyroid antibody (TPOAb and TgAb) levels. Results for TSH and thyroid hormones were conflicting. Some studies have noted a decrease or no change in TSH levels after vitamin D supplementation, while thyroid hormones remained mostly unchanged after vitamin D supplementation (Table 2). However, most of these studies were underpowered, with only four studies including more than 100 individuals [111,112,113,114]. The study with the largest number of participants included 11,017 participants in a wellness program receiving vitamin D supplementation, of whom 2% had hypothyroidism and 22% had subclinical hypothyroidism. Researchers observed a significant decrease in TPOAb, TgAb, TSH, thyroid hormone, and thyroglobulin levels in participants after 12 months of vitamin D supplementation [111]. Moreover, the number of patients with clinical and subclinical hypothyroidism significantly decreased after 12 months of vitamin D supplementation [111].

#### 5.2.3. Mendelian Randomization

Only two studies until now have used Mendelian randomization (MR) methodology to analyze the association between vitamin D and thyroid function. Ye et al., analyzed the association between serum vitamin D levels with 106 diseases/traits in 326,409 UK Biobank (UKBB) Europeans using MR analysis [128]. Using MR analysis, they did not observe a significant association between genetically predicted serum vitamin D levels and the risk of thyroid cancer, hypothyroidism and hyperthyroidism [128]. In the study that included 10,636 participants from China, Chen et al., using MR analysis, observed a causal relationship between genetically predicted decreased serum vitamin D levels and increased concentration of TPOAb [129]. However, genetically predicted TPOAb levels did not show an association with serum vitamin D levels [129]. Considering the importance of vitamin D for normal thyroid function, additional studies assessing the causal relationship between vitamin D and thyroid function using MR methodology, conducted in different ethnic groups, are of utmost importance.

## 6. Conclusions

In this review, we provided insight into the relationship between vitamin D status and thyroid function, including studies conducted only in humans. Although considerable progress has been made in elucidating the effect of vitamin D on thyroid function, it is still difficult to draw a definitive conclusion on how vitamin D affects thyroid function due to the high variability between the studies. Even though many studies have correlated 25(OH)D levels with the levels of TSH, thyroid hormones, and anti-thyroid antibodies (Table 1, Figure 1), there is still a large variability in results between studies. Studies in healthy participants and in participants with thyroid cancer observed either a negative correlation or no association between TSH and 25(OH)D levels, while the results for thyroid hormones showed higher variability. Studies comparing anti-thyroid antibodies (TPOAb, TgAb, TSHRAb) with 25(OH)D levels mostly observed a negative association between anti-thyroid antibodies and 25(OH)D levels, but many studies also failed to observe such an association (Table 1). However, in almost all studies investigating the effect of vitamin D supplementation on thyroid function, it was observed that vitamin D supplementation causes a significant decrease in the levels of anti-thyroid antibodies TPOAb and TgAb (Table 2, Figure 1). Several factors could contribute to the large variability between the studies. These include the use of different assays for the measurement of serum 25(OH)D levels between the studies [130], and the possible confounding effects of age, sex, body-mass index, seasonality, smoking, and dietary habits on 25(OH)D levels that were not taken into account in all studies [131,132,133]. Given the important role of vitamin D in normal thyroid function, additional cross-sectional observational studies and randomized controlled trials with long follow-ups are needed to understand the complex background underlying the interaction between vitamin D and thyroid function. Moreover, since only a few studies until today have used MR methodology to assess the causal relationship between vitamin D and thyroid function, additional studies using such methodology are crucial. In fact, MR is revolutionising epidemiological research by removing confounding factors from analyses and establishing the directionality of the inferred associations.

## Figures and Tables

**Table 1:** Correlation of 25(OH)D with TSH, thyroid hormones, thyroglobulin and anti-thyroid antibodies.

Reference	Correlation of 25(OH)D with TSH, Thyroid Hormones and Thyroglobulin	Correlation of 25(OH)D with Anti-Thyroid Antibodies	Number of Participants	Diagnosis of Participants
[56]	↔Thyroid function (levels of TSH, fT4, fT3)	↓iTgAb, ↓iTPOAb (i-isolated)	1812	Healthy controls
[57]		↓TPOAb	642	Healthy controls
[52]	↔TSH, ↔fT4	↓TPOAb	4181	Healthy controls
[54]	↔TSH, ↓fT3, ↓fT4		300	Healthy controls
[55]	↓TSH, ↔fT4, ↔fT3	↓TPOAb, ↓TgAb	1424	Adults (41–78 years)
[58]	↑TSH, ↔fT4, ↔fT3	↓TPOAb, ↔TgAb	155	Healthy controls
[51]	↔TSH, ↔fT4, ↑fT3	↓TPOAb, ↔TgAb	168	Elderly participants (65 years and older)
[59]	↔TSH, ↔fT4	↔TPOAb, ↔TgAb	2006	Healthy controls
[49]	↓TSH (also measured fT4 and fT3, but did not compare with 25(OH)D)		294	Healthy controls
[50]	↓TSH (only in younger participants)	↔TPOAb, ↔TgAb	2582	Healthy controls
[53]	↔TSH, ↑fT4, ↔fT3		123	Healthy controls
[60]	↔TSH, ↔fT4, ↔fT3		2869	Children (6–24 months of age)
[61]	↓TSH, ↑T3, ↑T4, ↑fT4	↓anti-thyroid antibodies (TPOAb, TgAb)	153	Pediatric cohort with balanced bone metabolism
[62]	↓TSH, ↔fT4, ↔fT3	(also measured TPOAb and TgAb, but did not compare with 25(OH)D)	261	Overweight subjects (216 patients with autoimmune thyroiditis)
[63]	↔TSH (also measured fT4 and fT3, but did not compare with 25(OH)D)		219	Obese Chinese people (118 with mildly increased TSH)
[64]	↓TSH, ↑fT4, ↑fT3	↔TPOAb, ↔TgAb	5262	Healthy controls (4889) and patients with Hashimoto’s thyroiditis (373)
[65]	↓TSH (in patients with Hashimoto’s thyroiditis), ↔fT4, ↔T4, ↔T3	↔TPOAb, ↔TgAb	637	Healthy controls (176) and patients with Hashimoto’s thyroiditis (461)
[66]	↔TSH	↑TPOAb (in males), ↔TgAb	185	Patients with Hashimoto’s thyroiditis (97) and healthy controls (88)
[67]	↔TSH, ↔fT4, ↔fT3	↓TPOAb ↓TgAb	39	Euthyroid women with Hashimoto’s thyroiditis
[68]	(also measured TSH and fT4, but did not compare with 25(OH)D)	↓TPOAb (in children with Hashimoto’s thyroiditis)	152	Children with Hashimoto’s thyroiditis (78) and healthy controls (74)
[69]	(also measured TSH, fT4 and fT3, but did not compare with 25(OH)D)	↔TPOAb, ↔TgAb	160	Hypothyroid patients with and without Hashimoto’s thyroiditis
[70]	↓TSH, ↔fT4		353	Patients with autoimmune thyroiditis (30%), multinodular goiter (21.81%), Basedow disease (1.98%), postoperative myxedema (6.52%) and other pathologies like single thyroid nodule or partial agenesia (the rest of the patients)
[71]	↔TSH, ↔fT4, ↔fT3	↓TPOAb (during winter, but not during summer)	933	Autoimmune thyroiditis
[72]	↔TSH, ↔fT4, ↔fT3	↓TPOAb, ↓TgAb	34	Autoimmune thyroiditis (women)
[73]	↔TSH, ↔fT4, ↔tT4, ↔fT3, ↔tT3	↓TPOAb, ↓TgAb	32	Prediabetic women with Hashimoto’s thyroiditis
[74]	↓TSH (in men, *n* = 2193), ↔fT4	↓TPOAb (in women, *n* = 2163)	4356	Euthyroid participants, euthyroid participants with TPOAb, participants with hypothyroidism
[75]	↔TSH, ↑fT4 (in patients with Hashimoto’s thyroiditis)	↔TSHAb, ↔TPOAb ↔TgAb	159	Patients with Hashimoto’s thyroiditis (88) and control subjects (71)
	↔fT4, ↔TSH in control group			
[76]	↔ TSH, ↔ fT4, ↔ fT3	↓TPOAb	200	Patients with Hashimoto’s Thyroiditis (100) and heathy euthyroid controls (100)
[77]	↔T4, ↔T3		21	Hyperthyroid patients
[78]	↔TSH, ↔fT4, ↔fT3	↔TPOAb, ↔TgAb	226	Patients with Graves’ disease (51), euthyroid Hashimoto’s thyroiditis (61), Hashimoto’s thyroiditis receiving hormone therapy (63) and healthy controls (51)
[79]	↓TSH, ↔fT4	↔TPOAb, ↔TgAb, ↔TSHRAb	776	Patients with Graves’ disease (148), Hashimoto’s thyroiditis (221) and participants with normal thyroid function and negative thyroid autoantibodies (407)
[80]	↔TSH, ↔fT4		224	Patients with diagnosed or suspected thyroid disease (hypo- and hyperthyroidism, thyroid nodule, and/or cancer)
[81]	↓TSH (in patients with Hashimoto’s thyroiditis) (also measured T4 and T3, but did not compare with 25(OH)D)	↔TPOAb	86	Patients with hypothyroid Hashimoto’s thyroiditis (41) and healthy euthyroid persons (45)
[82]	↔TSH (also measured fT4 and TgAb, but did not compare with 25(OH)D)	↔TPOAb	136	Children with Hashimoto’s thyroiditis (68) and healthy children (68)
[83]	↔TSH, ↔fT4, ↔fT3	↓TPOAb, ↔TgAb	394	Patients with Hashimoto’s thyroiditis (194) and healthy controls (200)
[84]	↓TSH, ↔fT4, ↑tT4, ↔fT3, ↔tT3	↔TPOAb, ↔TgAb	169	Patients with hypothyroid Hashimoto’s thyroiditis (90) and healthy controls (79)
[85]	↔TSH (also measured fT4 and fT3, but did not compare with 25(OH)D)	↔TPOAb, ↔TgAb, ↓TSHRAb	2 case control studies: (1) 210 (2) 171	2 case control studies: (1) Patients with Graves’ disease (70), Hashimoto’s thyroiditis (70) and healthy controls (70) (2) Women with post-partum thyroiditis (57) and euthyroid mothers as controls (114)
[86]	(also measured TSH, fT4 and fT3, but did not compare with 25(OH)D)	↓TPOAb (in patients with autoimmune thyroid disorder), ↔TSHRAb	304	Patients with autoimmune thyroid disorder (111) and without autoimmune thyroid disorder (193)
[87]	↔TSH, ↔T4, ↔T3		25	Infants with congenital hypothyroidism
[88]	↓fT3 (also measured TSH, fT4, tT4 and tT3, but did not compare with 25(OH)D)		108	Patients with hyperthyroidisms (55) and healthy controls (53)
[89]	(also measured TSH, fT4 and T3, but did not compare with 25(OH)D)	↔TBII, ↔TSAb	143	Patients with Graves’ disease
[90]	↔ TSH, ↔ fT4, ↔ fT3	↑ TSHRAb	188	Patients with Graves’ disease who received radioiodine therapy (128) and healthy controls (60)
[91]	↔TSH, ↔fT4, ↔fT3	↓TSHRAb, ↔TPOAb, ↔TgAb	140	Patients with Graves’ disease (70) and healthy controls (70)
[92]	↔ Thyroid function (described by the levels of TSH, tT4, tT3 and TPOAb)		398	Healthy controls (109) and patients with thyroid nodules (289)
[93]	↓TSH, ↔Tg	↔TgAb	1161	Patients with papillary thyroid cancer
[94]	↔TSH		548	Female patients with papillary thyroid cancer
[95]	↓TSH	↓TPOAb	820	Patients with papillary thyroid cancer
[41]	↔TSH		433	Patients with benign thyroid nodules and thyroid carcinomas
[96]	↓TSH, ↔fT4, ↑fT3		1706	Patients with papillary thyroid carcinoma (1578) and benign thyroid diseases (128)
[97]	↓TSH, ↔fT4, ↔fT3	(also measured TPOAb, TSHRAb and TgAb, but did not compare with 25(OH)D)	567	Patients with type 2 diabetes mellitus (389) and healthy controls (178)
[98]	↔TSH, ↔fT4		151	Patients with metabolic disorders
[99]	↓TSH, ↑fT4, ↑fT3	↓TPOAb, ↓TgAb	59	Women with post-partum thyroiditis; hypothyroid (14), euthyroid with post-partum thyroiditis (14), with non-autoimmune hypothyroidism (16) and healthy controls (15)
[100]	↔TSH, ↓fT3, ↔fT4	↔TPOAb, ↔TgAb	283	Pregnant women with vitamin D deficiency
[101]	↔TSH, ↔fT4, ↔fT3		132	Women in early pregnancy (1st trimester)
[102]	↔TSH, ↔fT4, ↔fT3	↔TPOAb, ↔TgAb	50	Pregnant women
[103]	↑TSH, ↓fT4, ↔tT4, ↓fT3, ↔tT3		277	Women in 2nd trimester of pregnancy
[104]	↓TSH, ↔fT4	↓TPOAb, ↓TgAb	200	Pregnant woman with subclinical hypothyroidism and gestational diabetes mellitus (100) and healthy pregnant woman (100)
[105]	↔TSH, ↔fT4, ↔fT3	↔TPOAb, ↔TgAb	50	Women with polycystic ovary syndrome (autoimmune thyroid disease detected in 12 patients)

This table includes only studies conducted in human subjects. (↔) no association, (↓) negative association, (↑) positive association. 25(OH)D, 25-hydroxyvitamin D; fT3, free triiodothyronine; fT4, free thyroxine; T3, triiodothyronine; T4, thyroxine; TBII, TSH-binding inhibitory immunoglobulin; Tg, thyroglobulin; TgAb, anti-thyroglobulin antibody; TPOAb, anti-thyroid peroxidase antibody; TSAb, thyroid-stimulating antibody; TSH, thyroid stimulating hormone; TSHRAb; TSH receptor antibody; tT3, total T3; tT4, total T4.

**Table 2:** Changes in the levels of TSH, thyroid hormones, thyroglobulin and anti-thyroid antibodies following vitamin D (cholecalciferol) therapy/supplementation.

Reference	Vitamin D Therapy/Supplementation Caused the Following Changes in the Levels of TSH, Thyroid Hormones and Thyroglobulin:	Vitamin D Therapy/Supplementation Caused the Following Changes in the Levels of Anti-Thyroid Antibodies:	Number of Participants	Diagnosis of Participants
[114] (meta-analysis)	↔ TSH, ↔ fT4, ↔ fT3	↓TPOAb, ↔TgAb	258	Hashimoto’s thyroiditis
[111]	↓TSH, ↓fT3, ↓fT4, ↓Tg	↓TPOAb, ↓TgAb	11,017	Participants in wellness program receiving vitamin D supplementation (2% hypothyroid and 22% subclinical hypothyroid)
[113]	↓TSH (in autoimmune thyroiditis positive group)		198	Autoimmune thyroiditis negative (103) and autoimmune thyroiditis positive (95)
[115]	↔TSH, ↔fT4	↓TPOAb	100	Patients with autoimmune thyroid disorder
[116]	↔TSH	↔TPOAb, ↔TgAb	34	Female patients with Hashimoto’s thyroiditis
[112]	↓TSH, ↔T4, ↔T3		201	Hypothyroid patients
[117]	Vitamin D/selenomethionine combination therapy caused: ↔TSH, ↔fT4, ↔fT3, ↓fT4/fT3	Vitamin D/selenomethionine combination therapy caused: ↓TPOAb, ↓TgAb	38	Euthyroid women with Hashimoto’s thyroiditis
[118]	Vitamin D/dehydroepiandrosterone(DHEA) combination therapy caused: ↓TSH, ↔fT4, ↔fT3	Vitamin D therapy or vitamin D/dehydroepiandrosterone (DHEA) combination therapy caused: ↓TPOAb, ↓TgAb	35	Women with Hashimoto’s thyroiditis
[119]	↔TSH, ↔fT4, ↔fT3, ↔fT4/fT3	↓TPOAb, ↓TgAb	62	Women with Hashimoto’s thyroiditis
[120]	↔TSH	↔TPOAb	56	Hashimoto’s thyroiditis
[121]	↓TSH, ↑T4		12	Hypothyroid patients
[122]	↔TSH, ↔fT4, ↔fT3	↓TPOAb, ↓TgAb	59	Non-lactating L-thyroxine-treated women with postpartum thyroiditis (38) and matched healthy postpartum women (21)
[123]	↔TSH, ↔fT4, ↔fT3	↓TPOAb, ↓TgAb	57	Levothyroxine-treated euthyroid women with Hashimoto’s thyroiditis and vitamin D insufficiency
[124]	↔TSH, ↔fT4, ↔fT3	↓TPOAb, ↓TgAb	34	Women with Hashimoto’s thyroiditis
[125]	↔TSH, ↔fT4, ↔fT3	↓TPOAb, ↓TgAb	37	Euthyroid men with autoimmune thyroiditis
[126]	↔TSH, ↔fT4, ↔fT3	↓TPOAb, ↓TgAb	36	Men with euthyroid Hashimoto’s thyroiditis and testosterone deficiency
[73]	Vitamin D/metformin combination therapy caused: ↓TSH, ↔fT4, ↔fT3	Vitamin D/ metformin combination therapy caused: ↓TPOAb, ↓TgAb	32	Women with Hashimoto’s thyroiditis
[127]	↓TSH (in patients receiving vitamin D supplementation), ↔T4, ↔T3	↔TPOAb, ↓TgAb (in patients receiving vitamin D supplementation)	40	Female patients with Hashimoto’s thyroiditis

This table includes only studies conducted in human subjects. (↔) no effect, (↓) decrease, (↑) increase. fT3, free triiodothyronine; fT4, free thyroxine; T3, triiodothyronine; T4, thyroxine; Tg, thyroglobulin; TgAb, anti-thyroglobulin antibody; TPOAb, anti-thyroid peroxidase antibody; TSH, thyroid stimulating hormone; tT3, total T3; tT4, total T4.

## Data Availability

Not applicable.

## Abbreviations

1,25(OH)2D, 1α, 25-dihydroxyvitamin D; 25(OH)D, 25-hydroxyvitamin D; Dio1, type 1 iodothyronine deiodinase; Dio2, type 2 iodothyronine deiodinase; fT3, free triiodothyronine; fT4, free thyroxine; HPT axis, Hypothalamus-pituitary-thyroid axis; MR, Mendelian randomization; NIS, sodium/iodide symporter; RXR, retinoid X receptor; T3, triiodothyronine; T4, thyroxine; TBII, TSH-binding inhibitory immunoglobulin; Tg, thyroglobulin; TgAb, anti-thyroglobulin antibody; TPO, thyroid peroxidase; TPOAb, anti-thyroid peroxidase antibody; TRH, thyrotropin-releasing hormone; TSAb, thyroid-stimulating antibody; TSH, thyroid stimulating hormone; TSHRAb; TSH receptor antibody; tT3, total T3; tT4, total T4; VDR, vitamin D receptor.
